# Partial versus Complete Fundoplication for the Correction of Pediatric GERD: A Systematic Review and Meta-Analysis

**DOI:** 10.1371/journal.pone.0112417

**Published:** 2014-11-11

**Authors:** Peter Glen, Michaël Chassé, Mary-Anne Doyle, Ahmed Nasr, Dean A. Fergusson

**Affiliations:** 1 University of Ottawa, Clinical Epidemiology Program, Ottawa Hospital Research Institute, The Ottawa Hospital, Ottawa, Ontario, Canada; 2 Clinical Epidemiology Program, Ottawa Hospital Research Institute, Ottawa, Ontario, Canada; 3 Department of Medicine, The Ottawa Hospital, Ottawa, Ontario, Canada; 4 Pediatric Surgeon, Division of General Surgery, Children’s Hospital of Eastern Ontario, Ottawa, Ontario, Canada; The Chinese University of Hong Kong, Hong Kong

## Abstract

**Background:**

There is no consensus as to what extent of “wrap” is required in a fundoplication for correction of gastroesophageal reflux disease (GERD).

**Objective:**

To evaluate if a complete (360 degree) or partial fundoplication gives better control of GERD.

**Methods:**

A systematic search of MEDLINE and Scopus identified interventional and observational studies of fundoplication in children. Screening identified those comparing techniques. The primary outcome was recurrence of GERD following surgery. Dysphagia and complications were secondary outcomes of interest. Meta-analysis was performed when appropriate. Study quality was assessed using the Cochrane Risk of Bias Tool.

**Results:**

2289 abstracts were screened, yielding 2 randomized controlled trials (RCTs) and 12 retrospective cohort studies. The RCTs were pooled. There was no difference in surgical success between partial and complete fundoplication, OR 1.33 [0.67,2.66]. In the 12 cohort studies, 3 (25%) used an objective assessment of the surgery, one of which showed improved outcomes with complete fundoplication. Twenty-five different complications were reported; common were dysphagia and gas-bloat syndrome. Overall study quality was poor.

**Conclusions:**

The comparison of partial fundoplication with complete fundoplication warrants further study. The evidence does not demonstrate superiority of one technique. The lack of high quality RCTs and the methodological heterogeneity of observational studies limits a powerful meta-analysis.

## Introduction

Gastroesophageal reflux disease (GERD) results from pathologic reflux of stomach content into the esophagus, causing troublesome symptoms [1]. In children the severity of symptoms is highly variable, ranging from epigastric pain to life threatening aspiration [2]. Management options reflect the variation in disease severity with mild cases responding well to medical therapy; however in severe or refractory cases surgical intervention is warranted [3].

Surgical fundoplication was first described in the adult literature in 1956 by Nissen [4]. This eponymous intervention creates an anatomic barrier to reflux by wrapping the gastric fundus around the esophagus in a complete 360 degree fashion [5], [6]. Contemporaries of Nissen described techniques involving a partial wrap. Thal and Toupet described wrapping the fundus partially (180 to 270 degrees) around the esophagus [7], [8]. All procedures have been incorporated into practices worldwide along with other derivations [9]–[11]. In the era of minimally invasive surgery, laparoscopic fundoplication in children has become accepted standard of practice [12]–[14].

While the benefit of laparoscopy is well accepted, the choice of a partial or complete wrap remains controversial. Advocates of the complete wrap claim better correction of GERD; conversely proponents of partial wrap point to lower rates of dysphagia and gas-bloating. A recent meta-analysis in adults found evidence to suggest correction of GERD similar for both techniques and higher rates of dysphagia and gas-bloat resulted from complete fundoplication [15]. The conclusion in this study was that partial fundoplication was the superior technique; however recent guidelines do not explicitly endorse this conclusion [16].

The adult literature cannot be expressly translated to management of children, as paediatric GERD is associated with different pathophysiology and is often associated with neurologic disorders. In children there is little evidence to guide the choice of wrap and current guidelines suggest both are acceptable [2], [17]. Although a systematic review conducted in 2011 concluded that there was equivalent control of reflux symptoms, it employed a strategy of pooling observational data with RCT data which can introduce bias [18]. Additionally, the study used did not use a comprehensive search strategy, reported a limited quality assessment, and may have missed important evidence. These factors support the need for an updated systematic review.

Our objective was to conduct a systematic review of interventional and observational studies comparing partial and complete fundoplication for pediatric GERD. This review was designed to compare reflux control, dysphagia, and complication rates between the two techniques. Additionally, we sought to ascertain the quality of the studies addressing the question.

## Materials and Methods

### Study Selection

A systematic literature search strategy using text and MeSH terms was used to conduct a search of the Medline (from 1946) and Scopus (from 1960) databases on February 15, 2013. The search was performed with no language or date restrictions (File S1 and File S2).

### Inclusion Criteria

Interventional and observational studies were eligible if they were designed to directly compare the outcomes of partial and complete fundoplication for the correction of gastroesophageal reflux disease (GERD). Partial fundoplication was defined as any surgical technique employing a wrap of less than 360 degrees to correct reflux disease. Complete fundoplication, commonly known as a Nissen fundoplication, was defined by a wrap of at least 360 degrees. Primary research studies involving children aged less than 18 years were included. Only studies that reported the success of anti-reflux surgery as measured by recurrence of the signs and symptoms of GERD were included. In studies for which there were multiple publications, the most recent publication was used. Only studies published in English or French were eligible.

### Exclusion Criteria

Studies where the pediatric population was indistinguishable from adults were eliminated. Animal studies, case reports, review articles, and editorials were ineligible. Studies in which the surgical indication was not GERD were also excluded (e.g. achalasia and congenital diaphragmatic hernia).

### Assessment of Study Eligibility

A two-stage screening of abstracts was performed to identify eligible studies. In Phase I, a standardized abstract screening tool was validated by three reviewers (PG, MC, and MD). This tool was used to eliminate ineligible studies by their titles and abstracts by the primary reviewer (PG). If there was uncertainty from the title or abstract then the article was kept for full text review. The abstracts kept after Phase I were reviewed by two reviewers in Phase II. Full text review and data abstraction were performed by PG, and MC or MD for all eligible studies.

### Outcomes

The primary outcome was the success rate of fundoplication to correct symptoms of GERD. A successful surgery was defined as one which corrected the signs and symptoms of GERD (i.e. an absence of pre-operative signs and symptoms). Determination of surgical success was grouped according to whether there was an objective method (eg. radiographic contrast studies, mannometry, 24 hour-pH monitoring, or endoscopic) or subjective method (patient reported, or investigator reported outcomes) of assessment.

Rates of dysphagia following fundoplication and other reported complications of the surgery were evaluated as secondary outcomes.

### Data Abstraction

The full text articles for studies with eligible abstracts were independently reviewed by the primary author (PG) and one other reviewer (MC or MD). Data from studies meeting inclusion criteria were captured using a standardized form which was tested using a pilot process. Study design elements, patient demographics, surgical techniques and definitions of surgical success were abstracted. Conflicts were resolved by consensus between the reviewers. In circumstances where the data was incomplete, an attempt was made to contact the primary author.

### Data Analysis

For the purposes of this review, all surgical techniques that employed a wrap of less than 360 degrees were grouped as a partial fundoplication. These were compared to subjects who had undergone a complete fundoplication. A pre-specified sub-group analysis was planned to assess the impact of neurological impairment on outcome. All data analysis was performed using the RevMan 5 software.

The results were analyzed separately for interventional and observational studies. Results were reported as Odds Ratios with 95% confidence intervals. We performed a meta-analysis of the interventional studies using a random effects model to calculate pooled odds ratio and 95% confidence interval. Statistical heterogeneity was measured using the I^2^ statistic.

### Risk of Bias

Each study was independently assessed for risk of bias by two reviewers using the Cochrane Risk of Bias tool [19]. Conflicts were resolved by consensus. The tool was modified to assess for the risk of bias from confounding factors which were not accounted for in the design or statistical analysis, this was evaluated within the “other” category of the tool.

### Review Protocol

This review was designed prior to conduct of the study, however there was no protocol registered.

## Results

### Search Results

**Figure 1:**
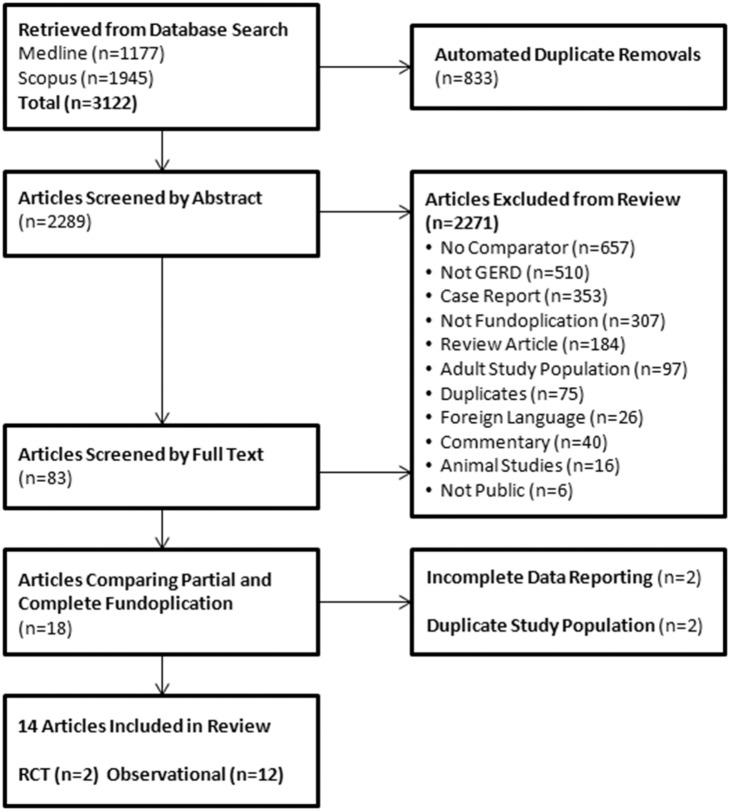
Flow chart of study selection process identifying studies comparing partial and complete fundoplication.

Our systematic search strategy yielded 2289 abstracts after automated removal of duplicates. Phase I of screening identified 83 potentially eligible articles; Phase II identified 14 studies to be included in the final analysis: 2 randomized controlled trials (RCT) [20], [21] and 12 retrospective cohort studies [22]–[33] (Figure 1).

### Study Characteristics

The included studies were published between 1994 and 2011. A total of 1927 subjects were identified, 967 (50%) were given a partial fundoplication. Patient age ranged from 2 months to 21 years (Table 1). Patient selection was based primarily on symptoms of GERD which were refractory to medical therapy (Table 1). Both RCTs required documented GERD on a pH study or upper gastrointestinal series (UGIS) before patient enrollment. Ten cohort studies required an objective measure of GERD, with one requiring only severe symptoms refractory to medical therapy [25]. In both RCTs and in 10 of the cohort studies a gastrostomy tube was a co-intervention for select patients.

**Table 1:** Study and patient characteristics for studies comparing partial and complete fundoplication.

Study (Year)	Study Design	Indication for Surgery	Partial Wrap Technique (n)	Complete Wrap Technique (n)	Co-Intervention	Length of Follow Up (Months)	Definition of Failed Reflux Surgery	NI patients Included in study	Median Age Age Range (years)
**Allal (2001)**	RC	Refractory GERD +/− UGIS +/−24 pH	Toupet (56)	Nissen (13) Nissen-Rosetti (70)	+/− Gastrostomy	10	GERD Symptoms + UGIS or pH	Y	NR 0.25–18
**Ceriati (1998)**	RC	Refractory GERD + UGIS or EGD +/− pH	Thal (20)	Nissen (27)	+/− Gastrostomy +/− Pyloroplasty	12–108	GERD Symptoms	Y (all)	5.3 0.5–20
**Cohen (1999)**	RC	Refractory GERD + UGIS +/− pH or EGD	Boix-Ochoa (27)	Nissen (32)	+/− Gastrostomy	6–216	GERD Symptoms	Y	NR 0.05–9
**Durante (2007)**	RCT	Encephalopathy Refractory GERD + pH study	Vertical Gastric Plication (7)	Nissen (7)	+/− Gastrostomy	6	GERD Symptoms, Clinical Score (not described)	Y (all)	NR 0.3–12.3
**Georgeson (1998)**	RC	Refractory GERD or severe pulmonary symptoms	Toupet (201)	Nissen (175)	+/− Gastrostomy	NR	GERD symptoms requiring surgical revision or medical therapy	NR	NR NR
**Goessler (2007)**	RC	Refractory GERD +/− pH or EGD Manometry or UGIS	Thal (24) Toupet (4)	Nissen (13)	+/− Gastrostomy +/− Pyloroplasty	6–47	GERD Symptoms requiring surgical revision or medical therapy	Y (all)	9.6 0.1–19
**Kazerooni (1994)**	RC	Clinical GERD +/− pH or EGD Manometry or UGIS	Thal (33)	Nissen (126)	NR	1–186	GERD Symptoms requiring surgical revision	Y	NR NR − 2
**Kubiak (2011)**	RCT	Refractory GERD + pH or EGD or UGIS	Thal (86)	Nissen (89)	+/− Gastrostomy	1–109	GERD Symptoms requiring surgical revision or medical therapy	Y	3.1 0.1–21
**Levin (2011)**	RC	Prior EA repair and presence of GERD symptoms	Toupet (18) Thal (5) Other (8)	Nissen (16)	+/− Gastrostomy +/− Hiatal Repair	12–213	GERD Symptoms recorded following EA repair	N	NR NR
**Strecker- McGraw (1998)**	RC	pH study	Thal (195)	Nissen (135)	+/− Pyloroplasty	3	Persistent abnormal pH study	Y	0.25 0.02–15
**Subramaniam (2000)**	RC	GERD Symptoms UGIS +/− EGD +/− pH Study	Boix-Ochoa (53)	Nissen (56)	+/− Gastrostomy	19–76	GERD Symptoms requiring surgical revision or medical therapy	Y	NR 1.5–11
**van der Zee (1994)**	RC	Refractory GERD + UGIS, pH, EGD	Thal (21)	Nissen (23)	+/− Gastrostomy	6–108	Persistent Esophagitis	Y	NR 0.2–15.6
**Wagener (2007)**	RC	Refractory GERD +/− EGD or pH or UGIS	Watson (55)	Nissen (76)	+/− Gastrostomy	0–90	Poor or Bad outcome, determined by surgeon	Y	2.6 0.05–16.9
**Weber (1999)**	RC	Refractory GERD +/− UGIS or EGD or pH	Toupet (154)	Nissen (102)	NR	12–96	Symptoms confirmed by UGIS +/− EGD or pH	N	NR 0.25–16

*RC* Retrospective Cohort; *RCT* Randomized Control Trial; *GERD* Gastroesophageal Reflux Disease; *UGIS* Upper Gastrointestinal Series; *pH* Twenty-Four Hour pH study; *EGD* Esophagoscopy; *EA* Esophageal Atresia; *NI* Neurologically Impaired; *Y* Yes; *N* No; *NR* Not Recorded.

The longest recorded follow up was 18 years in one study [24].

In all 14 studies the Nissen fundoplication was used as the complete wrap (Table 1). 12 of 14 studies used a single standard technique of partial fundoplication; 2 of 14 studies used at least 2 different partial wrap techniques. The most common partial fundoplications were the Thal (7/14 studies) [21], [23], [26]–[29], [31] and Toupet (5/14) [22], [25], [26], [28], [33]. The other partial techniques used included the Watson (1/14) [32], Boix-Ochoa (2/14) [24], [30], and Vertical Gastric Plication (1/14) [20].

### Primary Outcome: Correction of Reflux

**Figure 2:**
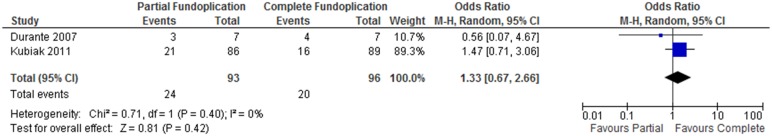
Recurrence of reflux symptoms following partial versus complete fundoplication (RCT).

Two randomized trials compared the performance of partial versus complete wraps. Kubiak et al. (2011) compared the Thal and Nissen wraps [21]. Follow-up for these patients ranged from 1–109 months. In this study the intervention was deemed successful if there was no need for anti-reflux medications or surgical revision. The RCT published by Durante et al. (2007) compared Vertical Gastric Plication with a Nissen wrap [20]. In this study success was determined using a clinical scoring system. Neither of these trials demonstrated a statistically significant difference between surgical techniques independently or in the pooled analysis, OR 1.33 [0.67, 2.66] (Figure 2).

Three of the twelve cohort studies objective measures to determine recurrence of symptoms. Symptoms of recurrence were confirmed using one or more of a pH study, upper gastrointestinal series (UGIS), or esophagogastroduodenoscopy (EGD). In the remaining 9 studies only patient or surgeon reported outcomes were used to measure recurrence.

**Figure 3:**
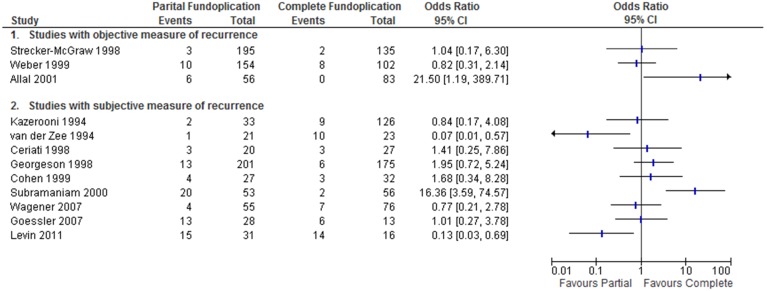
Recurrence of GERD following partial versus complete fundoplication (observational studies).

Two studies showed greater benefit from the complete fundoplication, while 2 other studies demonstrated greater benefit from the partial fundoplication when odds ratios were calculated from crude data (Figure 3). The remaining 8 studies did not demonstrate a significant difference between the two techniques. All studies compared the two techniques, but none reported adjusted odds ratios.

#### Subgroup Analysis of Neurologically Impaired Patients

**Figure 4:**
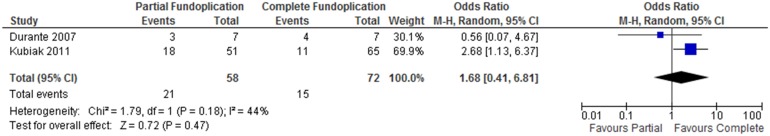
Recurrence of GERD following partial versus complete fundoplication in neurologically impaired patient subgroup (RCT).

Both RCTs enrolled a proportion of neurologically impaired patients [20], [21]. The pooled OR of these trials favoured success with complete fundoplication but did not demonstrate a statistically significant difference with OR 1.68 [0.41, 6.81] (Figure 4).

Nine observational studies enrolled some patients with neurological impairment and four reported specific outcomes for the neurologically impaired patients. There was insufficient evidence in each individual study to conclude a difference in symptomatic relief with either wrap.

### Secondary Outcome: Complications

A total of 25 different types of complications were reported in the 14 studies with the most commonly reported being dysphagia, gas-bloat syndrome, wrap disruption, and paraesophageal hernia (Table 2). Death was reported as a complication in 11 studies. All of these studies stated that death resulted from complications of an underlying chronic disease or were due to complications relating to gastrostomy tube displacement. There were no deaths attributed to the wrap technique.

**Table 2:** Reported complications, and number of patients affected for each fundoplication technique.

Complication	Kazerooni	Van der Zee	Ceriati	Georgeson	Strecker	Cohen	Weber	Subramamiam	Allal	Durante	Goessler	Wagener	Kubiak	Levin
Death	24 RNL	C: 1 P: 2	C: 1 P: 0	2 RNL		C: 1 P: 5		6 RNL	1 RNL	C: 1 P: 1	1 RNL	C:12 P: 5	C:21 P:10	
Dysphagia				C: 2 P: 5	2 RNL		C:20 P: 7	C: 3 P: 0	C: 7 P: 4			C: 6 P: 1	C:20 P:18	C: 6 P:14
Gas-Bloat Syndrome	C: 8 P: 2	C: 1 P: 0			3 RNL	C: 5 P: 0	RNL	2 RNL	C: 3 P: 0					
Paraesophageal Hernia		C:12 P: 0				C: 2 P: 0		C: 1 P: 0		C: 0 P: 1	6 RNL		C: 2 P: 5	C: 5 P: 4
Wrap Disruption	6 RNL	C: 3 P: 0		C: 2 P: 5	2 RNL						1 RNL	C: 1 P: 1	C: 2 P: 6	
Wrap Migration	2 RNL				1 RNL	C: 1 P: 0		C: 0 P: 2	3 RNL	C: 1 P: 0				
Small Bowel Obstruction		C: 1 P: 1				C: 6 P: 0		2 RNL			1 RNL	C: 1 P: 2		
Pneumonia						C: 1 P: 0		1 RNL		C: 0 P: 2	6 RNL	C: 5 P: 1		
Wrap Stricture		C: 3 P: 0							1 RNL					C: 3 P: 5
Wound Dehiscence						C: 1 P: 0		1 RNL	1 RNL					
Infection						C: 1 P: 0						C: 2 P: 1		
Regurgitation						C: 1 P: 0								C: 4 P: 6
Atelectasis						C: 1 P: 1					1 RNL			
Gastric Perforation				1 RNL		C: 1 P: 0								
Hematoma								1 RNL	1 RNL					
Peritonitis	1 RNL							1 RNL						
Pneumothorax									1 RNL			C: 1 P: 0		
Retching												C: 5 P: 1		C: 4 P: 3
Bleeding									2RNL					
Esophageal Perforation				1 RNL										
Food Bolus												C: 1 P: 0		
Gastric Torsion											1 RNL			
Intussusception						C: 1 P: 0								
Not Specified			C: 6 P: 4											
Spleen Laceration												C: 1 P: 0		

*C:* Complete Fundoplication; *P*: Partial Fundoplication; *RNL*: Reported, Not Listed by Fundoplication Type.

#### Postoperative Dysphagia

**Figure 5:**
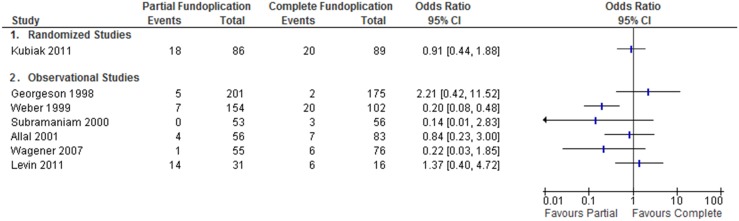
Dysphagia following partial versus complete fundoplication.

Dysphagia was reported in one interventional study. This study did not find a difference in incidence of dysphagia between the two techniques [21] (Figure 5).

Seven observational studies reported post-operative dysphagia. The definition and severity of dysphagia varied between studies. One found a significant benefit of the partial fundoplication on rates of dysphagia, OR 0.20 [0.08,0.48] [33] (Figure 5). In this study, patients were selected to receive partial fundoplication if it was felt there was underlying esophageal or gastric dysmotility. This confounder was not accounted for in the original publication. Additionally, there was no distinction made between dysphagia and gas-bloat syndrome in this publication. Attempts to reach the author for further clarification were unsuccessful.

### Trial Quality Assessment

**Figure 6:**
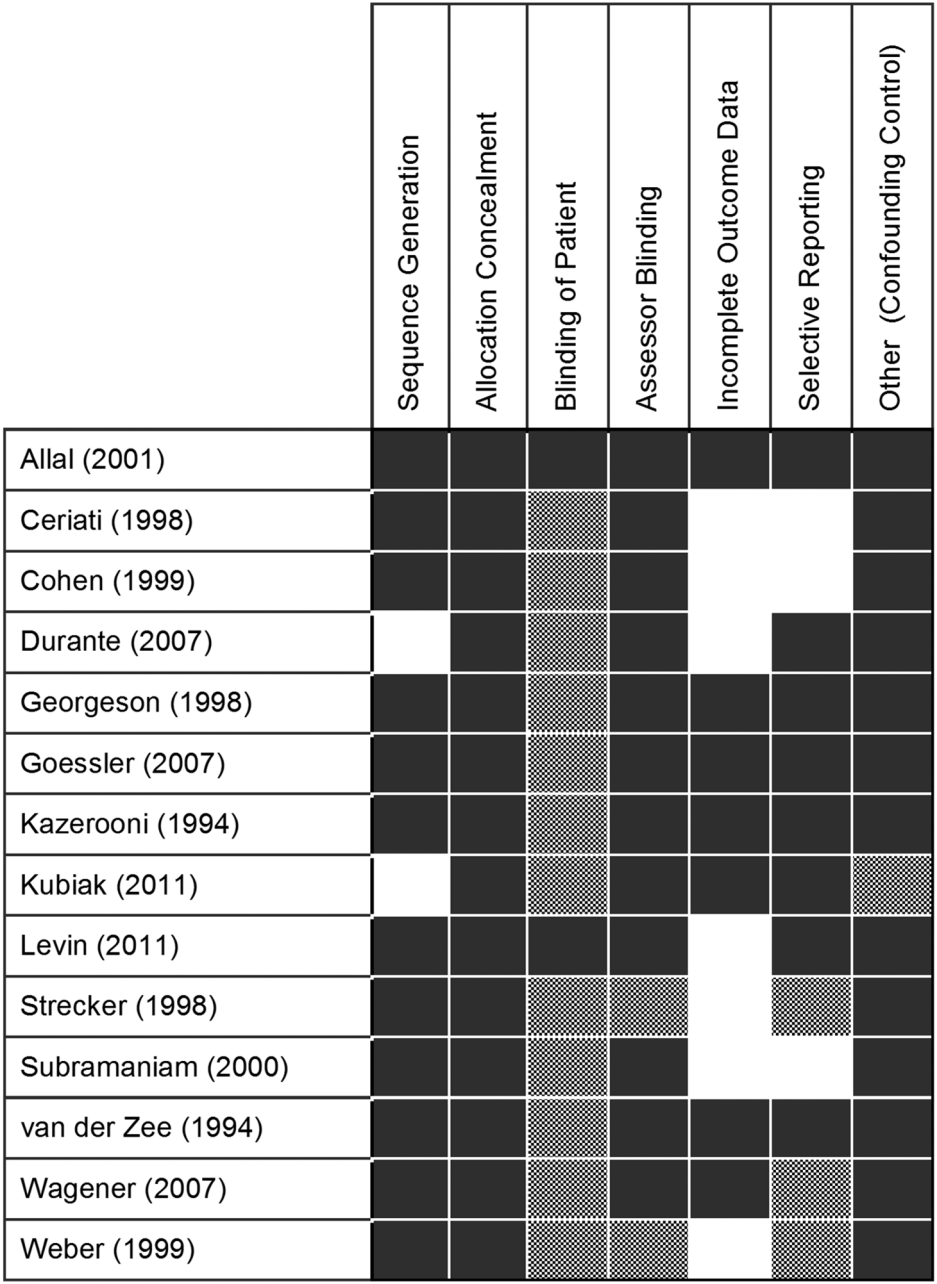
Modified Cochrane Risk of Bias evaluation of study quality. Shaded: high risk of bias; checkered: unclear or indeterminate risk of bias; not shaded: low risk of bias.

Results of the Cochrane Risk of Bias demonstrate that overall trial quality was poor (Figure 6). The RCT published by Durante scored a low risk of bias on two of the domains, unclear on one, and high in the remaining four domains. The study published by Kubiak et al. was at higher risk of bias with three unclear and four high risk domains.

The observational studies scored similarly. With respect to the control of confounding factors, all studies scored a high risk of bias as it was unclear if any control for confounding factors had been conducted.

## Discussion

Surgical fundoplication for correction of GERD refractive to medical therapy is a standard intervention. The results of this review find no evidence to indicate that the extent of wrap, partial or complete, results in a superior correction of GERD symptoms. The paucity of well conducted studies in this area justifies for further study. The lack of well conducted RCTs is a problem not only for the study of this question, but many pediatric surgical questions [34].

In a previous review addressing the question of complete versus partial fundoplication, trials were pooled irrespective of their trial design [18]. This may lead to biased results as consideration for study design is vital when pooling non-randomized trial data [35]. We chose to distinguish between these two designs, and pool only RCTs. There was great variation between studies with respect to their definition of GERD, definition of treatment failure, and length of follow up (Table 1). These factors further affect the ability to draw meaningful conclusions from pooled data.

High quality RCTs are vital to providing evidence-based estimates of effectiveness and safety to clinicians [36]. The quality of trials designed to study pediatric fundoplication are on the whole poor (Figure 6). Only in the domain of completeness of outcome reporting did at least half of the trials demonstrate low risk of bias (7/14). Understanding these high risks of bias are important for understanding the limitations of this review and state of the evidence.

Our primary outcome was correction of GERD following partial and complete fundoplication. There were two RCTs designed to answer this question. The study by Durante used a symptom score to grade the recurrence of reflux [20], however there was no description given for this score. The study by Kubiak et al. was based on symptom recurrence requiring medical therapy or surgical revision [21]. With these differences in mind, we conducted a pooled analysis for the primary outcome. There was no statistical difference in correction of GERD following partial or complete fundoplication (Figure 2). We also did not find a difference in outcome in the neurologically impaired subgroup (Figure 3).

In the observational trials, heterogeneity was even more apparent (Table 1). Indications for surgery were based on a variety of tests and symptom scores; likewise there was variation in the definitions of failed surgery. Outcome reporting in these studies was generally poorly done, with none of these trials performing any statistical control for confounders. The trial heterogeneity and statistical analysis precludes any meaningful meta-analysis, as the reported information is insufficient to satisfy the Cochrane criteria for inclusion of non-randomized studies [37]. Instead, we have chosen to present the data as a Forest plot based on an intention to treat analysis conducted using the data presented in this group of trials. Figure 4 shows that there is great variation in the conclusions drawn from each of these trials, confirming the notion that there are uncontrolled confounders driving this data.

Heterogeneous trials presented similar issues for the analysis of our secondary outcome measure: post-operative dysphagia. The review published by Mauritz et al. concluded that there was a greater incidence of post-operative dysphagia following complete fundoplication [18]. We did not reach the same conclusion as we could not find a reliable means of pooling the outcomes addressing this question (Figure 5).

The variety of post-operative complications found in these studies is indicative both of the morbidity associated with the surgery and with underlying medical conditions often necessitating fundoplication. We have employed a systematic strategy to identify complications arising from surgery and shown that there are a large number of potential complications which both the surgeon and patient should be aware of. Table 2 outlines 25 post-operative complications identified across the 14 studies. Some of these certainly overlap and are indicative of synonymous description of symptoms (e.g. retching and regurgitation). It should also be noted that for many studies there was concomitant placement of gastrostomy tubes. This intervention was typically added to fundoplication in cases of severe neurologic impairment. This understanding is important when interpreting the complications. For instance, 11 trials reported mortality data, however in all of these trials death was attributed to the underlying neurologic impairment or complications attributed to the gastrostomy tube [20]–[27], [30], [32]. This spectrum of possible complications should be understood by clinicians proposing to manage GERD with anti-reflux surgery.

For surgeons who are planning fundoplication for a patient the evidence presented here suggests that an individualized treatment plan be developed. Both techniques are technically similar, with the main variation being the placement of intra-corporeal sutures. Patient factors are thus the variables which should drive the decision on the extent of wrap required. In children these factors will vary with age and severity of symptoms, as well as the tolerance for post-operative issues with dysphagia. Although the evidence is poor, we would suggest that partial fundoplication should be considered for patients in whom tolerance for post-operative dysphagia is low. If the primary concern is correction of reflux, and there is little concern for the effects of possible dysphagia, then complete Nissen fundoplication should be the intervention of choice.

Further study on fundoplication technique in children is warranted. Currently there is one study registered with clinicaltrials.gov which have not yet been reported in the literature (NCT 00480285). This study was due to have been completed in 2011 yet no publication was found. Further interventional trial research is indicated to define which technique is better able to control reflux symptoms. Research to identify pre-operative factors associated with treatment complications, such as dysphagia, would also be beneficial.

## Conclusion

The comparison of partial fundoplication with complete fundoplication warrants further study. Choosing between partial and complete fundoplication will rely mainly on the preferences of the surgeon and patient as presently there is no conclusive evidence to support one technique over another. The lack of high quality RCTs in the area points to the need for a well conducted and reported study, as there is insufficient evidence to establish a conclusive answer using meta-analysis. The variation in the definition of GERD failure also suggests that a universal definition be defined.

## Supporting Information

Checklist S1 **PRISMA checklist.**
(DOCX)

File S1 **Search string for Medline database.**
(DOCX)

File S2 **Search string for Scopus database.**
(DOCX)
