# Supplementary material for: Partial versus Complete Fundoplication for the Correction of Pediatric GERD: A Systematic Review and Meta-Analysis
Source: PLoS One. 2014 Nov 11;9(11):e112417. doi: 10.1371/journal.pone.0112417 (PMC4227692; doi:10.1371/journal.pone.0112417)
Supplement: File S1 — Search string for Medline database. (DOCX) [file pone.0112417.s002.docx]

**S.1** Search string for Medline database.

1 exp Gastroesophageal Reflux/ (20519)

2 gerd.tw. (4933)

3 gastroesophageal reflux.tw. (12524)

4 gastro oesophageal reflux.tw. (3364)

5 gastro esophageal reflux.tw. (995)

6 gastric acid reflux.tw. (36)

7 esophageal reflux.tw. (1480)

8 or/1-7 (25325)

9 Fundoplication/ (3290)

10 fundoplication.tw. (4107)

11 nissen.tw. (2332)

12 Belsey.tw. (186)

13 Toupet.tw. (358)

14 Dor.tw. (1490)

15 (hemifundoplicat$ or hemi fundoplicat$).tw. (49)

16 or/9-15 (6694)

17 8 and 16 (3625)

18 adolescent/ or exp child/ or exp infant/ (2692174)

19 (child$ or adolescen$ or infant$ or baby or babies).tw. (1225699)

20 Pediatrics/ (37629)

21 p?ediatric$.tw. (196474)

22 or/18-21 (2968512)

23 17 and 22 (1177)
