# Supplementary material for: Partial versus Complete Fundoplication for the Correction of Pediatric GERD: A Systematic Review and Meta-Analysis
Source: PLoS One. 2014 Nov 11;9(11):e112417. doi: 10.1371/journal.pone.0112417 (PMC4227692; doi:10.1371/journal.pone.0112417)
Supplement: File S2 — Search string for Scopus database. (DOCX) [file pone.0112417.s003.docx]

**S.2** Search string for Scopus database.

((TITLE-ABS-KEY(fundoplication) OR TITLE-ABS-KEY(belsey) OR TITLE-ABS-KEY(toupet) OR TITLE-ABS-KEY(hemifundoplication) OR TITLE-ABS-KEY(hemifundoplication))) **AND**

((TITLE-ABS-KEY(child) OR TITLE-ABS-KEY(children) OR TITLE-ABS-KEY(infant) OR TITLE-ABS-KEY(infants) OR TITLE-ABS-KEY(baby) OR TITLE-ABS-KEY(babies) OR TITLE-ABS-KEY(pediatric) OR TITLE-ABS-KEY(paediatric) OR TITLE-ABS-KEY(pediatrics) OR TITLE-ABS-KEY(paediatrics)))

(1945)
